# Desmoteplase for Acute Ischemic Stroke within 3 to 9 Hours after Symptom Onset: Evidence from Randomized Controlled Trials

**DOI:** 10.1038/srep33989

**Published:** 2016-09-27

**Authors:** Ligen Shi, Feng Liang, Yunping Li, Anwen Shao, Keren Zhou, Jun Yu, Jianmin Zhang

**Affiliations:** 1 Department of Neurosurgery, Second Affiliated Hospital, School of Medicine, Zhejiang University, Hangzhou, Zhejiang, China; 2 Brain Research Institute, Zhejiang University, Hangzhou, Zhejiang, China; 3 Collaborative Innovation Center for Brain Science, Zhejiang University, Hangzhou, Zhejiang, China

## Abstract

Recent studies have shown inconsistent results regarding the value of desmoteplase for treating acute ischemic stroke (AIS) when administered within an extended time window. We performed a meta-analysis to explore the value of desmoteplase in AIS treatment. The MEDLINE, EMBASE, and Cochrane Library databases were searched for randomized controlled trials (RCTs) that had evaluated desmoteplase versus placebo for AIS. The primary outcomes were intracranial hemorrhage (ICH) within 72 hours and favorable outcome at Day 90. We pooled 819 patients from 5 RCTs. Desmoteplase treatment showed a neutral effect on favorable outcome (*P* = 0.42) but a favorable safety profile in terms of ICH (*P* = 0.64) compared with the placebo group. In the subgroup analysis, 90 μg/kg desmoteplase, a late time to treatment (6–9 hours), and serious stroke symptoms at baseline (NIHSS > 12) subgroups showed high risks of ICH (*P* ≤ 0.02). A high dose of desmoteplase (125 μg/kg) showed a tendency to improve recanalization (*P* = 0.05), but was also associated with an increased risk of death (*P* = 0.04). In conclusion, desmoteplase administered over an extended time window had no significant effect on functional recovery but exhibited a favorable safety profile in patients with AIS.

Worldwide, acute ischemic stroke (AIS) is a life-threatening illness with high mortality and substantial disability, and involves large financial burdens^1^. The use of intravenous alteplase, the only approved thrombolytic agent for AIS, is restricted to within 3 hours of symptom onset^2^. Although, its use has recently been extended to up to 4.5 hours in most European countries^3^, the window of treatment opportunity remains narrow. In addition, intravenous alteplase is associated with increased risks of bleeding, especially fatal intracranial hemorrhage (ICH)^3^. Hence, there is an unmet need for new thrombolytic drugs with better safety and efficacy profiles for AIS over an extended time window.

Desmoteplase, a clot-dissolving protein containing 441 amino acids, is regarded as an ideal thrombolytic agent for AIS^4^. Preclinical studies have indicated that desmoteplase has high fibrin specificity^5^, a long half-life^5^, a low bleeding tendency^6^, a lack of neurotoxicity^4^, and does not induce blood–brain barrier damage^6^. These positive results stimulated enthusiasm for the translation of research results to humans^7^,^8^,^9^,^10^,^11^. The first phase II trial (the Desmoteplase in Acute Ischemic Stroke Trial, DIAS) randomly assigned AIS patients (3 to 9 hours after stroke onset with 20% perfusion/diffusion mismatch) to receive fixed doses in the range of 25 to 50 mg, but was terminated due to excessive symptomatic ICH (sICH)^11^. The revised design with a lower weight-adjusted doses of desmoteplase (62.5, 90, or 125 μg/kg), showed a dose-dependent effect on recanalization after 4–8 hours of thrombolytic therapy and favorable outcomes after 3 months. Importantly, the results of the revised study showed a favorable safety profile in patients receiving desmoteplase^11^. The 62.5 μg/kg dose had no significant effect on recanalization^11^. Another phase II trial (Dose Escalation of Desmoteplase for Acute Ischemic Stroke, DEDAS) investigated the efficacy and safety of 90 or 125 μg/kg desmoteplase for AIS using a similar design as DIAS^10^. The results of DEDAS indicated that revascularization was improved with 125 μg/kg desmoteplase and treatment was strongly associated with good clinical outcome^10^. Unfortunately, findings from two further phase III trials, DIAS-2 and DIAS-3, showed that desmoteplase had no clinical efficacy on either recanalization after 4–8 hours or favorable outcome after 3 months^8^,^9^. Moreover, DIAS-2 reported an unexpected finding: a relatively high death rate was observed in the 125 μg/kg desmoteplase group^9^. Nonetheless, DIAS-2 was criticized because only 30% of the enrolled patients had an intracranial vessel occlusion^9^, and DIAS-3 was limited by the high rate of protocol violations^8^. A low dose-ranging design in Japanese patients provided a favorable tolerability and neutral efficacy in the 70 or 90 μg/kg desmoteplase group^7^. Recently, an on-going phase III trial, DIAS-4, selected patients with occlusion or high-grade stenosis in major cerebral arteries based on computed tomography angiography (CTA) or magnetic resonance angiography (MRA) and small ischemic infarcts based on computed tomography (CT) or magnetic resonance imaging (MRI), to assess the safety and efficacy of 90 μg/kg desmoteplase given 3–9 hours after the onset of ischemic stroke.

Based on the above-mentioned results from previous preclinical studies and clinical trials, the efficacy and safety of desmoteplase treatment for AIS over an extended time window are unclear. Several issues need to be resolved, including the optimal dosage of desmoteplase, and the appropriate imaging modality with which to select patients with salvageable penumbra. The present meta-analysis pooled data from previous clinical trials to investigate the value of desmoteplase treatment for AIS within 3–9 hours of symptom onset, and to explore the potential factors that might influence the efficacy and safety of desmoteplase.

## Methods

We performed this meta-analysis according to the preferred reporting items for systematic reviews and meta-analyses (PRISMA) format guidelines^12^.

### Search Strategy and Information Sources

Two investigators (LGS and FL) searched three major databases, MEDLINE, EMBASE, and the Cochrane Library, for relevant articles published between January 1980 and February 2016. The combination of the variables “desmoteplase” AND “ischemic stroke” were used to match the titles and abstracts in the MEDLINE database. The search strategy for EMBASE and the Cochrane Library were similar to that used for MEDLINE. In addition, two investigators (LGS and FL) also independently screened reference lists from randomized controlled trials (RCTs), post-hoc analyses, critiques, comments, meta-analyses, and reviews to ensure all relevant studies had been included in this study.

### Study Selection and Data Collection

We only included RCTs with AIS patients who had received desmoteplase within 3–9 hours of symptom onset. Specific eligibility criteria were as follows: (a) adult subjects (aged > 18 years); (b) follow-up information available for 3 months after treatment; (c) treatment with intravenous desmoteplase; (d) endpoints assessed functional recovery and ICH.

All records were evaluated by two authors (LGS and FL) in accordance with the eligibility criteria as mentioned above. The following data were extracted from the included RCTs after strict selection and evaluation: basic information on the included trials, eligibility criteria and study design, and outcome assessments (Table 1).

### Outcomes Definition and Quality Assessment

The primary efficacy endpoint was the favorable outcome of functional recovery, which was defined as a modified Rankin Scale (mRS) score of 0–2 or the combination of an improvement in the National Institutes of Health Stroke Scale (NIHSS) of ≥8 points from baseline (or NIHSS ≤ 1), an mRS score of 0–2, and a Barthel Index (BI) of 75–100 at Day 90. Secondary efficacy outcomes included recanalization within 24 hours and symptomatic cerebral edema within 72 hours. The primary safety endpoint was ICH, including composed of sICH and asymptomatic ICH (aICH). The definition of sICH was ICH with an increase in the NIHSS of ≥4 points at 72 hours. aICH was defined as any ICH that did not reach the criteria of sICH. Secondary safety outcomes included major hemorrhage within 72 hours, and death rate at Day 90.

The risk of bias in individual studies was assessed by two investigators (LGS and FL) using the Review Manager 5.2 software (Cochrane Collaboration, UK). We applied the uniform criteria of the Cochrane collaboration to assess the risk of bias in RCTs. These evaluative criteria included selection bias, performance bias, detection bias, attrition bias, reporting bias, and other potential biases.

### Data Synthesis and Analysis

Dichotomous outcomes were analyzed as the risk ratio (relative risk [RR]; 95% confidence interval [CI]) and calculated using a fixed-effects model. All data analyses were performed using STATA (Version 12.0). Statistical heterogeneity was estimated by the *I*^*2*^ statistic. Sensitivity analysis was used to detect the stability of the consolidated results. Publication bias was detected using Egger’s funnel plot with pseudo 95% confidence limits. The presence of publication bias was evaluated with a Begg–Mazumdar rank correlation test. Tests were two-tailed and a *P* value of less than 0.05 was considered to be significant for all analyses.

## Results

### Study Selection and Characteristics

**Figure 1:**
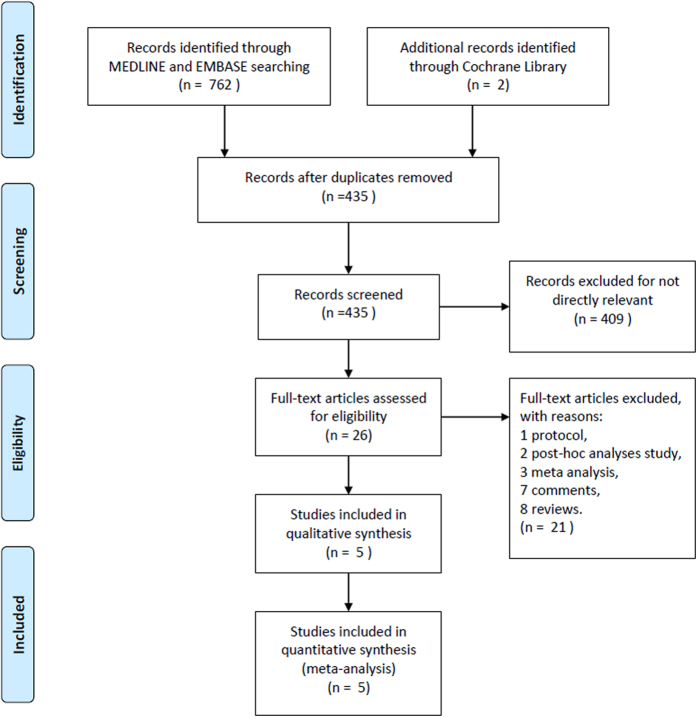
The study search, selection and inclusion process.

A total of 764 titles and abstracts were screened (Fig. 1). After removing the duplicates and irrelevant records, 26 full-text articles were assessed for eligibility. An additional 21 articles were excluded due to the limitation of publication types: 1 protocol study, 2 post-hoc analyses, 3 meta-analyses, 7 comments, and 8 reviews. Ultimately, we identified 5 articles that met the eligibility criteria for this meta-analysis^7^,^8^,^9^,^10^,^11^. Of the 5 articles included in the analysis, all except one^7^ were international multicenter trials. DIAS-2 and DIAS-3 were phase III clinical trials and were published in *Lancet Neurology*. The remaining three phase II clinical trials were published in *Stroke*. The detailed characteristics of the included studies are listed in Table 1.

### Efficacy and Safety Endpoints

**Figure 2:**
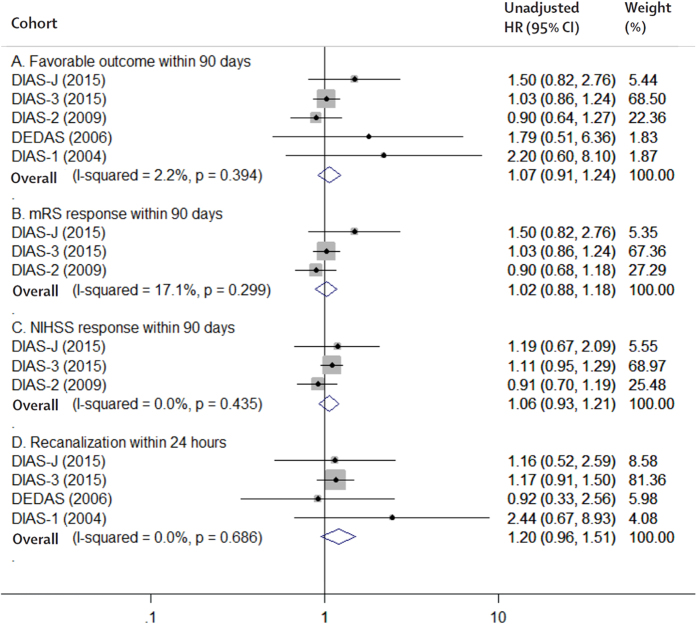
The pooled relative risk of the efficacy outcomes. The diamond indicates the estimated relative risk (95% confidence interval) for all patients.

**Figure 3:**
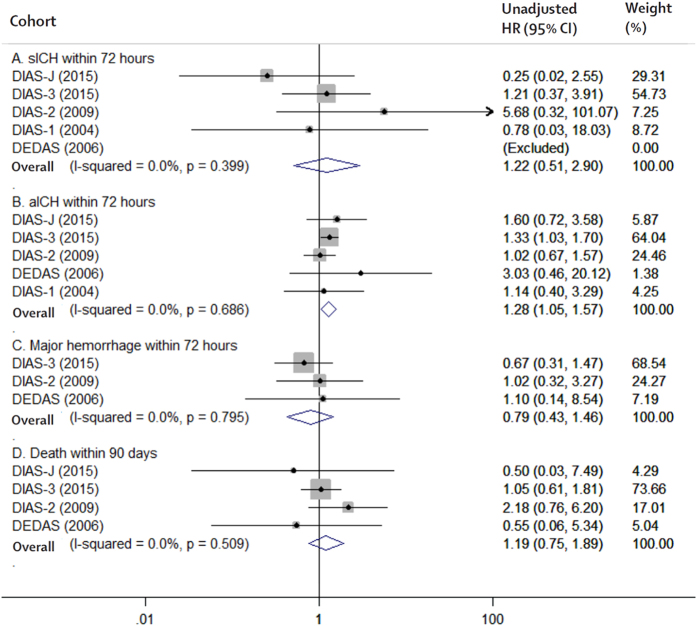
The pooled relative risk of the safety outcomes. The diamond indicates the estimated relative risk (95% confidence interval) for all patients.

All 5 RCTs enrolling 819 patients were available for the analysis of primary efficacy and safety outcomes. Desmoteplase showed a neutral effect on favorable outcome (RR 1.07, 95% CI 0.91 to 1.24, *P* = 0.42; Fig. 2A), a favorable safety profile in sICH (RR 1.22, 95% CI 0.51 to 2.90, *P* = 0.66; Fig. 3A), but an increased risk of aICH (RR 1.28, 95% CI 1.05 to 1.57, *P* = 0.02; Fig. 3B) compared with the placebo group. No significant differences were observed in any of the secondary efficacy or safety outcomes, including mRS response at Day 90 (*P* = 0.78; Fig. 2B), NIHSS response at Day 90 (*P* = 0.36; Fig. 2C), recanalization within 24 hours (*P* = 0.11; Fig. 2D), major hemorrhage within 72 hours (*P* = 0.46; Fig. 3C), or death rate at Day 90 (*P* = 0.45; Fig. 3D), between the desmoteplase and placebo groups.

### Subgroup and Sensitivity Analyses

Subgroup analyses were performed to examine the influence of desmoteplase dosage, infarct volume and stroke severity at baseline, time to treatment, and imaging modality. High dose of desmoteplase (125 μg/kg) showed a tendency to improve in recanalization (RR 2.25, 95% CI 1.02 to 5.01, *P* = 0.05; Table 2), but an increased risk of death (RR 2.66, 95% CI 1.04 to 6.83, *P* = 0.04; Table 2). Subgroups including 90 μg/kg desmoteplase, late time to treatment (6–9 hours), and serious stroke symptoms at baseline (NIHSS > 12), showed high risks of ICH (*P* ≤ 0.02; Table 2). The sensitivity analysis showed that all of the consolidated results were stable.

### Heterogeneities and Publication Bias

For all analyses pertaining to efficacy and acceptability, no evidence existed for the between-study of heterogeneities assessed by the Cochrane *I*^*2*^ statistic (data not shown). Publication bias was detected using Egger’s funnel plot with pseudo 95% confidence limits, which showed low risks (data not shown).

### Quality of the Included Studies

**Figure 4:**
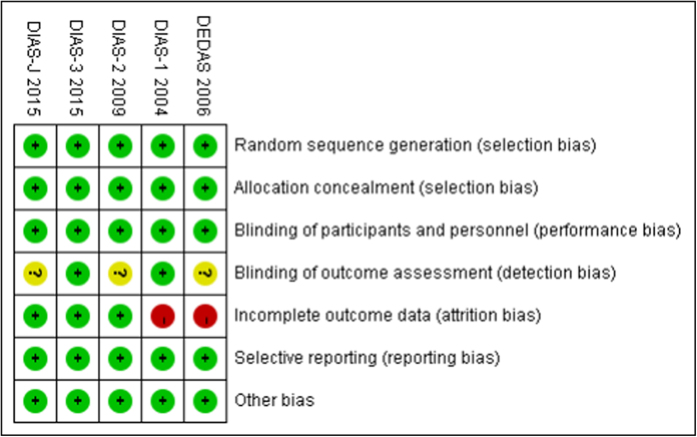
Risk of bias: A summary table for each risk of bias item for each study.

Details about the risks of bias of the included studies are shown in Fig. 4. Three trials did not give details about the blinding of outcome assessments (detection bias). Two trials lacked a detailed explanation of side effects, which might have led to high risks of incomplete outcome data (attrition bias).

## Discussion

Desmoteplase treatment for AIS over an extended time window might be questionable based on the evidence from our present meta-analysis. We found that desmoteplase had no significant benefit in any of the efficacy endpoints, including early recanalization and late functional recovery, but exhibited a favorable safety profile. Subgroup analyses indicated that high dose of desmoteplase could improve early recanalization, but could simultaneously increase the risk of mortality. In addition, serious stroke symptoms at baseline, defined as an NIHSS score of >12, administration of 90 μg/kg desmoteplase, and the initiation of treatment 6–9 hours after symptom onset, were high risk factors for ICH.

Thrombolytic therapy is based on the hypothesis that salvageable brain tissue could be restored after recanalization^13^. Hence, the achievement of a positive result from desmoteplase trials is based on three hypotheses: first, patients with salvageable penumbra could be accurately identified using multimodal MRI or CT^14^,^15^; second, desmoteplase is sufficiently effective to dissolve a thrombus in a late time window^16^; third, recanalization is associated with favorable functional outcomes^17^,^18^. The first important task is to distinguish those patients with salvageable brain tissue who might be sensitive to intravenous thrombolytic therapy. A mismatch hypothesis has been proposed in that a mismatch between perfusion- and diffusion-weighted MRI will predict the response to thrombolysis^19^. Previous studies have shown that a larger perfusion/diffusion mismatch size was predictive of a more favorable functional outcome in patients receiving intravenous alteplase after 3 hours^20^,^21^,^22^. The DEDAS trial also indicated that perfusion/diffusion mismatch was a more important predictor of clinical outcomes than the duration of symptoms in the 6- to 9-hour time window analysis^10^. Whether the salvageable penumbra can be measured reliably at multiple centers is unclear. Previous post-hoc analyses showed that imaging modality played a role in detecting the effect of therapy^23^,^24^. Small ischemic lesions less than 25 ml in volume based on diffusion-weighted MRI were associated with good reperfusion and favorable clinical outcomes in patients receiving intravenous alteplase for AIS within an extended time window^23^,^24^. However, no association was observed between CT-selected small ischemic lesions and good functional recovery^25^. Both CT and MRI were used to evaluate the perfusion/diffusion mismatch of the infarct volume in the included trials, which might influence the stability of the results from different trials and centers. In addition, the DIAS, DEDAS, and DIAS-2 trials defined the penumbra as at least a 20% mismatch in the infarct volume between perfusion- and diffusion-weighted image^9^,^10^,^11^. This definition was not a standard quantitative method of penumbra measurement^26^, which might partly explain the negative results of our present subgroup analysis of patients selected by perfusion/diffusion mismatch. Additional studies should focus on the accurate measurement of the salvageable penumbra to differentiate those patients who were still able to achieve a favorable recovery with intravenous thrombolytic therapy.

The core issue is whether desmoteplase could dissolve a thrombus to obtain a favorable functional outcome when administered within the late time window. There was no doubt that recanalization would lead to a favorable clinical outcome over an extended time window in patients with a large perfusion/diffusion mismatch. A previous post-hoc analysis demonstrated that recanalization was associated with a favorable functional outcome both in the desmoteplase group (*P* < 0.001) and in the placebo group (*P* < 0.027) in the 3- to 9-hour window^8^. From a pathophysiological point of view, the pharmacological efficacy of recanalization therapies for major intracranial artery occlusions might have a time-dependent ceiling effect^27^,^28^. Salvageable brain tissue might shrink with time due to the irreversible infarcted core enlargement after the onset of stroke symptoms^21^. Therefore, we performed a subgroup analysis to test the influence of the infarct core volume at baseline. The subgroup analysis showed no significant differences in any of the observed indicators, including favorable outcome, recanalization, ICH, and death, in either >25 ml or <25 ml ischemic lesion subgroups. The subgroup analysis of NIHSS scores also showed similar results. The NIHSS score was thought to correlate with infarct core volume^29^. The clots present in the late time window might be more resistant to thrombolysis than those present in the early time window^16^. In addition, cardioembolic clots are easier to recanalize with thrombolysis than those originating elsewhere^30^. However, none of the included trials have addressed the characteristics of the clots. We performed subgroup analyses to explore the influence of the time to treatment. However, we observed that both 6- to 9-hour and 3- to 6-hour treatment subgroups showed no significant efficacy of desmoteplase for AIS, but the 6- to 9-hour treatment subgroup had a higher incidence of ICH compared with the 3- to 6-hour subgroup. Some researchers have proposed that those patients arriving at the emergency ward during a late time window often had small infarct cores or excellent collateral circulation^27^. Better collateral circulation means smaller infarcts and larger mismatch volumes, which predict better functional recovery^5^,^31^. Those patients with good collateral circulation in the placebo group might influence the real effect of desmoteplase on recanalization.

The question of whether desmoteplase reached an appropriate concentration is unclear. Our present meta-analysis showed that the 125 μg/kg dose of desmoteplase led to a tendency to improve in recanalization (*P* = 0.05). The DIAS trial indicated that there was a dose-dependent effect of treatment with 62.5, 90, or 125 μg/kg desmoteplase on both recanalization (23.1, 46.7, and 71.4%) and favorable outcome (13.3, 46.7, and 60%, respectively)^11^. Additionally, a post-hoc analysis that excluded patients without a significant imaging mismatch and an internal carotid artery occlusion indicated that patients receiving 125 μg/kg desmoteplase experienced a better clinical outcome than those receiving placebo^10^. The present meta-analyses showed that 125 μg/kg of desmoteplase was associated with an increased incidence of death, but there was no significant difference between patients receiving 125 μg/kg desmoteplase and those receiving placebo after excluding deaths unrelated to the trial medicine. In addition, treatment with 125 μg/kg desmoteplase was not associated with a higher risk of ICH compared with the placebo group. Unfortunately, treatment with 125 μg/kg desmoteplase showed no significant impact on functional recovery at 3 months. Although the DIAS trial used a high fixed dose of desmoteplase between 25 mg (median 313 μg/kg) and 50 mg (median 546 μg/kg), a number of cases of sICH occurred. The exploration of an acceptable high dosage of desmoteplase based on the results of our present meta-analysis and previous studies mentioned above is still necessary.

Limitations in our analysis should be noted. First, we performed this meta-analysis based on limited data. Only 5 published RCTs with 819 patients were pooled to test the efficacy and safety of desmoteplase for AIS over an extended time window. The use of desmoteplase should be carefully in clinical practice, although Begg’s funnel plot data showed no publication bias in our present study. Second, the included trials showed heterogeneity in imaging modality. MRI was considered to be more sensitive than CT in detecting early ischemic infarcts^8^. The planned post-hoc subgroup analysis of the DIAS-3 trial indicated that desmoteplase treatment was associated with a significant improvement in patients with an infarct volume of less than 25 ml based on diffusion-weighted MRI, but not in CT-selected patients with small ischemic infarct volumes^8^. Unfortunately, we could not test this detection effect because we were unable to obtain the original data of the included trials. A meta-analysis of individual patient data should be performed in the future. Finally, the quality of the included studies was not ideal. The DIAS-2 was criticized for only including 30% of patients with an intracranial vessel occlusion^9^, and DIAS-3 was limited by the high rate of protocol violations^8^. Although the sensitivity analysis showed that all of the consolidated results were stable, these defects of the included studies should not be ignored.

In summary, our meta-analysis suggests that desmoteplase treatment had no objective efficacy on functional recovery but did exhibit a favorable safety profile in patients with AIS over an extended time window. A high dose of desmoteplase might exhibit potential efficacy and favorable safety results in recanalization, which therefore could improve the final functional recovery. Future trials should examine the potential therapeutic effect of desmoteplase at an acceptable high dose in patients with a larger perfusion/diffusion mismatch.

## Additional Information

**How to cite this article**: Shi, L. *et al.* Desmoteplase for Acute Ischemic Stroke within 3 to 9 Hours after Symptom Onset: Evidence from Randomized Controlled Trials. *Sci. Rep.* **6**, 33989; doi: 10.1038/srep33989 (2016).

## Figures and Tables

**Table 1:** Characteristics of the Included Studies and Outcome Events.

Trials	DIAS-J 2015 (NCT01104467)	DIAS-3 2015 (NCT00790920)	DIAS-2 2009 (NCT00111852)	DEDAS 2006 (NCT00638248)	DIAS-1 2004 (NCT00638781)
**1. Information of the Included Trials**					
*** Regions***	18 centers in Japan	77 centers in 17 countries	44 centers in 8 countries	25 centers in 2 countries	44 centers in 12 countries
*** Phases***	II	III	III	II	II
*** Journal***	Stroke	Lancet Neurology	Lancet Neurology	Stroke	Stroke
**2. Eligibility Criteria and Study Design**					
*** Inclusion Criteria***	Acute ischemic stroke;Time window: 3–9 hours;NIHSS: 4–24;Cerebral artery occlusion or high-grade stenosis in MCA.	Acute ischemic stroke;Time window: 3–9 hours;NIHSS: 4–24;Cerebral artery occlusion or high-grade stenosis in proximal cerebral arteries.	Acute ischemic stroke;Time window: 3–9 hours;NIHSS: 4–24;≥20% perfusion/diffusion mismatch.	Acute ischemic stroke;Time window: 3–9 hours;NIHSS: 4–20;≥20% perfusion/diffusion mismatch.	Acute ischemic stroke;Time window: 3–9 hours;NIHSS: 4–20;≥20% perfusion/diffusion mismatch.
*** Exclusion Criteria***	Infarction >1/3 of the MCA territory, ICH, etc.	Infarction >1/3 of the MCA territory, >1/2 ACA or PCA territory, ICH, etc.	Infarction >1/3 of the MCA territory, total ACA territory, ICH, etc.	Internal carotid artery occlusions, ICH, etc.	Infarction >1/3 of the MCA territory, ICH, etc.
*** Study Design***	Desmoteplase 70 μg/kg or 90 μg/kg vs. Placebo.	Desmoteplase 90 μg/kg vs. Placebo.	Desmoteplase 90 μg/kg or 125 μg/kg vs. Placebo.	Desmoteplase 90 μg/kg or 125 μg/kg vs. Placebo.	Desmoteplase 62.5 μg/kg, 90 μg/kg or 125 μg/kg vs. Placebo.
**3. Outcomes Assessments**					
*** Efficacy outcomes***	Favorable outcome, mRS response, change in NIHSS, and NIHSS response at 90 days; Recanalization at 12–24 hours.	Favorable outcome, mRS response, NIHSS response, and composite response at 90 days; Recanalization at 12–24 hours.	Favorable outcome, mRS response, NIHSS response, BI response, and composite response at 90 days; Change in infarct volume at 30 days.	Favorable outcome at 90 days; Recanalization at 4–8 hours; Change in infarct volume at 30 days	Favorable outcome at 90 days; Recanalization at 4–8 hours; Change in infarct volume at 30 days.
*** Safety outcomes***	sICH, aICH, any ICH, and SCE within 72 hours and 90 days; AEs, SAEs, and death at 90 days.	sICH, aICH, major hemorrhage, and SCE within 24 hours; AEs and death at 90 days.	sICH and aICH within 72 hours; AEs and death at 90 days.	sICH, aICH, and major hemorrhage within 72 hours; AEs, anaphylactic reaction, and death at 90 days.	sICH, aICH, and major hemorrhage within 72 hours; AEs, anaphylactic reaction, and death at 90 days.

**NIHSS**: National Institutes of Health Stroke Scale; **mRS**: modifed Rankin Scale; BI: Barthel Index; **MCA**: Middle Cerebral Artery; **ACA**: Anterior Cerebral Artery; **PCA**: Posterior Cerebral Artery; **SCE**: Symptomatic Cerebral Edema; **ICH**: Intracranial Hemorrhage; **ICH**: symptomatic Intracranial Hemorrhage; **aICH**: asymptomatic Intracranial Hemorrhage; **AE**: Adverse Events; **SAE**: Severe Adverse Events.

**Table 2:** Subgroup Analysis of Efficacy and Safety Outcomes.

	Efficacy outcomes				Safety outcomes			
	Favorable outcomes		Recanalization		ICH		Death	
	RR (95% CI)	P value	RR (95% CI)	P value	RR (95% CI)	P value	RR (95% CI)	P value
**1. Dose of desmoteplase**								
** *****62.5 *****μ*****g*****/*****kg***	0.73 (0.12, 4.43)	0.74	1.10 (0.22, 5.51)	0.91	1.22 (0.37, 4.06)	0.74	N/A	N/A
** *****70 *****μ*****g*****/*****kg***	1.43 (0.73, 2.80)	0.30	1.11 (0.45, 2.78)	0.82	0.73 (0.40, 1.31)	0.29	0.33 (0.01, 7.62)	0.49
** *****90 *****μ*****g*****/*****kg***	1.08 (0.92, 1.27)	0.33	1.17 (0.85, 1.60)	0.34	1.29 (1.05, 1.57)	0.01	1.00 (0.61, 1.64)	1.00
** *****125 *****μ*****g*****/*****kg***	1.08 (0.74, 1.56)	0.69	2.25 (1.02, 5.01)	0.05	1.28 (0.84, 1.96)	0.25	2.66 (1.04, 6.83)	0.04
**2. Infarct volume at baseline**								
** *****<25 ml***	1.01 (0.79, 1.28)	0.94	1.16 (0.52, 2.59)	0.73	1.20 (0.81, 1.78)	0.37	0.50 (0.03, 7.49)	0.62
** *****>25 ml***	1.12 (0.92, 1.35)	0.27	1.54 (0.68, 3.47)	0.30	1.35 (0.46, 3.93)	0.59	1.60 (0.52, 4.94)	0.41
**3. Stroke severity at baseline**								
** *****NIHSS***** > *****12***	1.11 (0.94, 1.32)	0.23	1.20 (0.96, 1.51)	0.11	1.32 (1.06, 1.64)	0.01	0.99 (0.59, 1.66)	0.97
** *****NIHSS***** < *****12***	0.90 (0.64, 1.27)	0.55	N/A	N/A	1.16 (0.72, 1.87)	0.55	2.18 (0.76, 6.20)	0.14
**4. Time to treatment**								
** *****>6 h***	1.04 (0.89, 1.22)	0.59	1.15 (0.91, 1.45)	0.23	1.30 (1.06, 1.59)	0.01	1.16 (0.73, 1.84)	0.54
** *****<6 h***	2.20 (0.60, 8.10)	0.24	2.44 (0.67, 8.93)	0.18	0.98 (0.27, 3.51)	0.97	N/A	N/A
**5. Imaging modality**								
** *****P*****/*****D mismatch***	1.06 (0.77, 1.46)	0.74	1.54 (0.68, 3.47)	0.30	0.55 (0.34, 0.87)	0.01	1.81 (0.71, 4.58)	0.21
** *****DWI infarct***	1.07 (0.90, 1.27)	0.46	1.17 (0.92, 1.48)	0.20	1.25 (0.99, 1.56)	0.06	1.02 (0.60, 1.74)	0.94

**ICH**: Intracranial Hemorrhage; **RR**: Relative Risk; **CI**: Confidence Interval; **P/D**: Perfusion/Diffusion; **DWI**: Diffusion-Weighted Imaging; **N/A**: Not Applicable.
